# Supplementary material for: Preclinical Evaluation of the Antimicrobial-Immunomodulatory Dual Action of Xenohormetic Molecules against Haemophilus influenzae Respiratory Infection
Source: Biomolecules. 2019 Dec 17;9(12):891. doi: 10.3390/biom9120891 (PMC6995536; doi:10.3390/biom9120891)
Supplement: Supplementary file 1 [file biomolecules-09-00891-s001.pdf]

Supplementary Material

Preclinical evaluation of the antimicrobial-immunomodulatory dual action of xenohormetic molecules against *Haemophilus influenzae* respiratory infection

Ariadna Fernández-Calvet[1+], Begoña Euba[1,2+], Lucía Caballero[1], Roberto Díez-Martínez[3], Margarita Menéndez[2,4], Carlos Ortiz de Solórzano[5,6], José Leiva[7,8], Vicente Micol[9,10], Enrique Barrajón-Catalán[9], Junkal Garmendia[1,2*]

[1]Instituto de Agrobiotecnología, CSIC-Gobierno Navarra, Mutilva, Spain; [2]Centro de Investigación Biomédica en Red de Enfermedades Respiratorias (CIBERES), Madrid, Spain; [3]Ikan Biotech SL, The Zebrafish Lab, Centro Europeo de Empresas e Innovación de Navarra (CEIN); [4]Instituto de Química Física Rocasolano, CSIC, Madrid, Spain; [5]Laboratory of Preclinical Models and Analytical Tools, Division of Solid Tumors and Biomarkers, Center for Applied Medical Research, Pamplona, Spain; [6]Centro de Investigación Biomédica en Red de Enfermedades Oncológicas (CIBERONC), Madrid, Spain; [7]Servicio de Microbiología, Clínica Universidad de Navarra, Pamplona, Spain; [8]Instituto de Investigación Sanitaria de Navarra (IdiSNA), Pamplona, Spain; [9]Instituto de Investigación, Desarrollo e Innovación en Biotecnología Sanitaria de Elche (IDiBE), Instituto de Biología Molecular y Celular (IBMC), Miguel Hernández University, Elche, Spain; [10]Centro de Investigación Biomédica en Red de Fisiopatología de la Obesidad y Nutrición (CIBERobn), Madrid, Spain

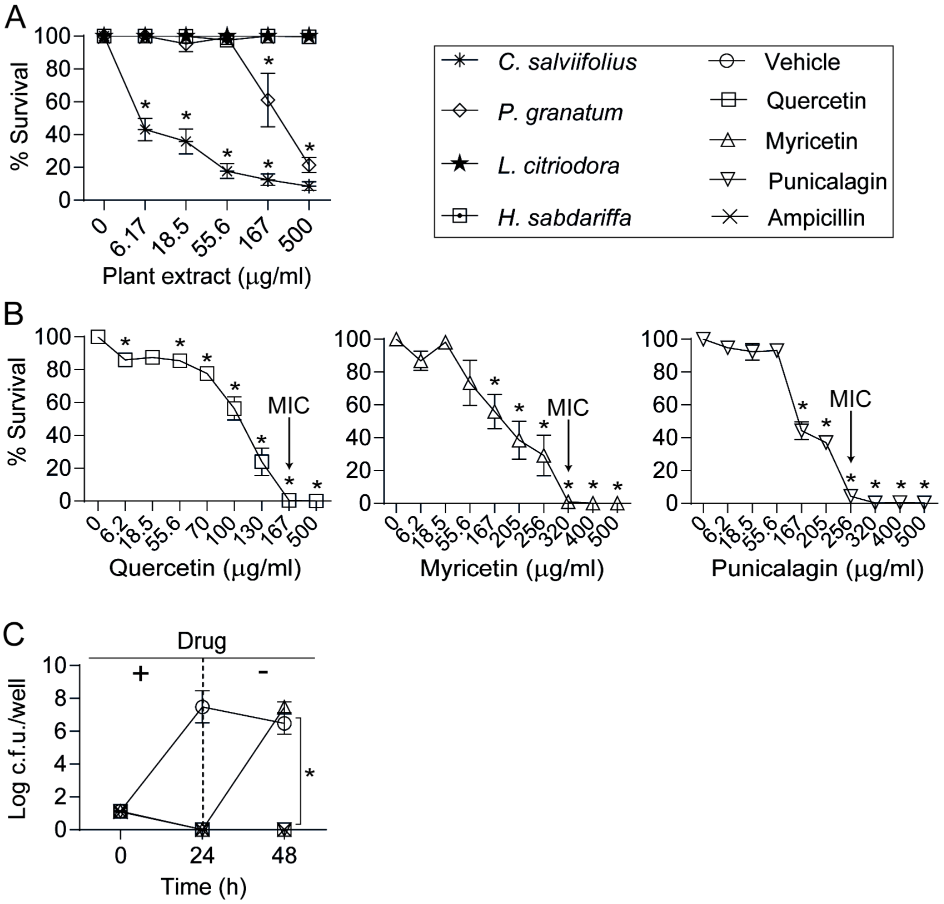

Figure S1. Antimicrobial effect of plant extracts and pure polyphenols on *H. influenzae* RdKW20. (**A**) RdKW0 strain is susceptible to *C. salviifolius* and *P. granatum* extracts in a dose-dependent manner (* P<0.005). (**B**) Polyphenols quercetin (*P<0.005), myricetin (*P<0.0001) and punicalagin (*P<0.0001), reduced RdKW20 survival in a dose-dependent manner. Survival percentage (mean ± SEM) is shown (**A** and **B**). (**C**) Quercetin and punicalagin have a bactericidal effect on RdKW20 strain, when comparing bacterial counts (log c.f.u./well, mean ± SD) after incubation with- and without polyphenol inhibitory concentrations. At 24 h, only bacteria incubated with vehicle control rendered counts, when compared with ampicillin-treated cultures (*P<0.0001). After polyphenol replacement by sBHI, 48 h bacterial cultures previously incubated with myricetin rendered significant counts, when compared with quercetin, punicalagin and ampicillin treated cultures (*P<0.0001). Statistical comparisons of the means were performed with two-way ANOVA (**A** and **C**) or one-way ANOVA (**B**), and Dunnett's multiple comparisons test.

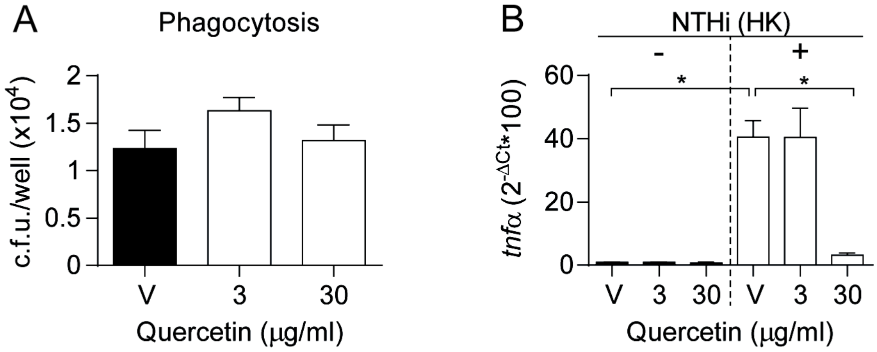

Figure S2. Quercetin has an anti-inflammatory effect on NTHi-infected alveolar macrophages. (**A**) For phagocytosis assays, murine alveolar macrophages (MH-S) were pretreated with quercetin or DMSO (vehicle solution, V) for 4 h. Drugs were removed prior infection. M-HS cells were infected with NTHi375 strain for 1 h, and incubated with gentamicin for 1 h more. No significant differences were observed in NTHi375 phagocytosis between quercetin- and vehicle-treated cells. Results are shown as c.f.u./well (mean ± SEM). (**B**) M-HS cells were pretreated with quercetin or DMSO for 4 h; next, heat killed (HK) bacteria were used as an inflammatory stimulus (NTHi HK) for 1 h (white bars). In parallel, non-stimulated cells were included as negative controls (black bars). Drugs were maintained throughout the assay. To monitor gene expression, relative quantity of murine *tnfα* was measured by qRT-PCR. Gene expression was significantly increased upon infection of vehicle-treated cells (*P<0.0001). Quercetin 30 µg/mL significantly decreased *tnfα* expression level (*P<0.0001). Results are shown as the relative gene expression levels (mean ± SEM). Statistical comparisons of the means were performed with one-way ANOVA and Bonferroni’s multiple comparisons test.

Table S1. Primers used for qRT-PCR in this study.

Primer name	Primer ID	Sequence (5'-3')	Organism	Reference
il8- qPCR-F	1241	AGAGACAGCAGAGCACAC	Human	(1)
il8- qPCR-R	1242	AGTTCTTTAGCACTCCTTGG	Human	(1)
gapdh-qPCR-F	1237	GAAGGTGAAGGTCGGAGTC	Human	(1)
gapdh-qPCR-R	1238	GAAGATGGTGATGGGATTTC	Human	(1)
cxcl1- qPCR-F	1247	GCAGGGAATTCACCCCAAGA	Human	(1)
cxcl1- qPCR-R	1248	CTTCAGGAACAGCCACCAGT	Human	(1)
pde4b- qPCR-F	1259	GAGACAAAGAGCGGGAGAGG	Human	(2)
pde4b- qPCR-R	1260	GGTGGTGAGGGACTTTGAGG	Human	(2)
il6- qPCR-F	1717	TAGTGAGGAACAAGCCAGAGC	Human	This study
il6- qPCR-R	1718	TTGGGTCAGGGGTGGTTATTG	Human	This study
kc- qPCR-F	1404	GACAGACTGCTCTGATGGCA	Mouse	(2)
kc- qPCR-R	1405	TGCACTTCTTTTCGCACAAC	Mouse	(2)
mtnfα- qPCR-F	1592	AGGCACTCCCCCAAAAGATG	Mouse	(2)
mtnfα - qPCR-R	1593	GCTCCTCCACTTGGTGGTTT	Mouse	(2)
mpde4b- qPCR-F	1753	TGGAAATCCTGGCTGCCAT	Mouse	This study
mpde4b - qPCR-R	1754	TCCACAGAAGCTGTGTGCT	Mouse	This study
mgapdh-qPCR-F	1430	CCCACTAACATCAAATGGGG	Mouse	(3)
mgapdh-qPCR-R	1431	CCTTCCACAATGCCAAAGTT	Mouse	(3)

Table S2. Minimal inhibitory concentration (MIC) of quercetin, myricetin and punicalagin against NTHi clinical isolates, measured by microdilution (µg/mL).

NTHi strain	$MIC_{quercetin}$	$MIC_{myricetin}$	$MIC_{punicalagin}$
P650	76.0 ± 12.7	312.5 ± 21.3	125.0
P657	122.9 ± 50.7	165.3 ± 69.1	250.0
P665	90.6 ± 47.6	192.3 ± 19.6	62.5

References

1. Euba B, Moleres J, Segura V, Viadas C, Morey P, Moranta D, Leiva J, de-Torres JP, Bengoechea JA, Garmendia J. 2015. Genome expression profiling-based identification and administration efficacy of host-directed antimicrobial drugs against respiratory infection by nontypeable *Haemophilus influenzae*. Antimicrob Agents Chemother 59:7581-92.
2. Euba B, Lopez-Lopez N, Rodriguez-Arce I, Fernandez-Calvet A, Barberan M, Caturla N, Marti S, Diez-Martinez R, Garmendia J. 2017. Resveratrol therapeutics combines both antimicrobial and immunomodulatory properties against respiratory infection by nontypeable *Haemophilus influenzae*. Sci Rep 7:12860.
3. Regueiro V, Moranta D, Frank CG, Larrarte E, Margareto J, March C, Garmendia J, Bengoechea JA. 2011. *Klebsiella pneumoniae* subverts the activation of inflammatory responses in a NOD1-dependent manner. Cell Microbiol 13:135-53.
